# l-Arginine intervention at hyper-acute phase protects the prolonged MRI abnormality in MELAS

**DOI:** 10.1007/s00415-016-8069-4

**Published:** 2016-06-22

**Authors:** Miyuki Kitamura, Shuichi Yatsuga, Toshi Abe, Nataliya Povalko, Reo Saiki, Kikumi Ushijima, Yushiro Yamashita, Yasutoshi Koga

**Affiliations:** 1 Department of Pediatrics and Child Health, Kurume University School of Medicine, 67 Asahi-Machi, Kurume, Fukuoka 830-0011 Japan; 2 Department of Radiology, Kurume University School of Medicine, 67 Asahi-Machi, Kurume, Fukuoka 830-0011 Japan; 3 Institute of Fundamental Medicine and Biology, OpenLab, Gene and Cell Technologies, Kazan Federal University, Kazan, 42000 Russia

Dear Sirs,

Though neurological signs in mitochondrial encephalopathy, lactic acidosis, and stroke-like episodes (MELAS) resemble features of ischemic stroke. According to the limited analyses, recurrent strokes are associated with vasogenic edema by diffusion weighted image (DWI) on magnetic resonance image (MRI) [1, 2]. Endothelial dysfunction has been found in MELAS [3], and l-arginine, a potent donor of nitric oxide at physiological condition, has been used as a therapeutic purpose [4]. In this study, we evaluate the therapeutic effects of l-arginine infusion at super-acute phase of stroke-like episodes (SLE) on the clinical and the serial neuroimaging analysis in two MELAS patients.

Patient 1 is a 7-year-old girl who was diagnosed as MELAS based on a finding of symptoms and 79 % mutation of an A3243G in the mitochondrial DNA in urine. She has a history of SLE at the age of 5.5 years-old. She complained of the sudden headache, vomit, hemi-convulsion and hemianopia in the left side. After getting the written informed consent (Kurume University IRB#9715), we infused 0.5 g/kg/dose of l-arginine within 60 min after the onset and took the brain MRI at 18 h from the onset. All of clinical symptoms disappeared within 30 min after l-arginine infusion without using anti-convulsants. The series of MRIs were performed till 6 month from the onset of stroke-like episodes. MRI obtained at 18 h after the onset showed high intensity signal in T2WI, and DWI and low in ADC in right and left hemisphere of cerebrum and right occipital region. However those abnormal findings completely normalized on MRIs at 7 days and 1 month later.

**Fig. 1:**
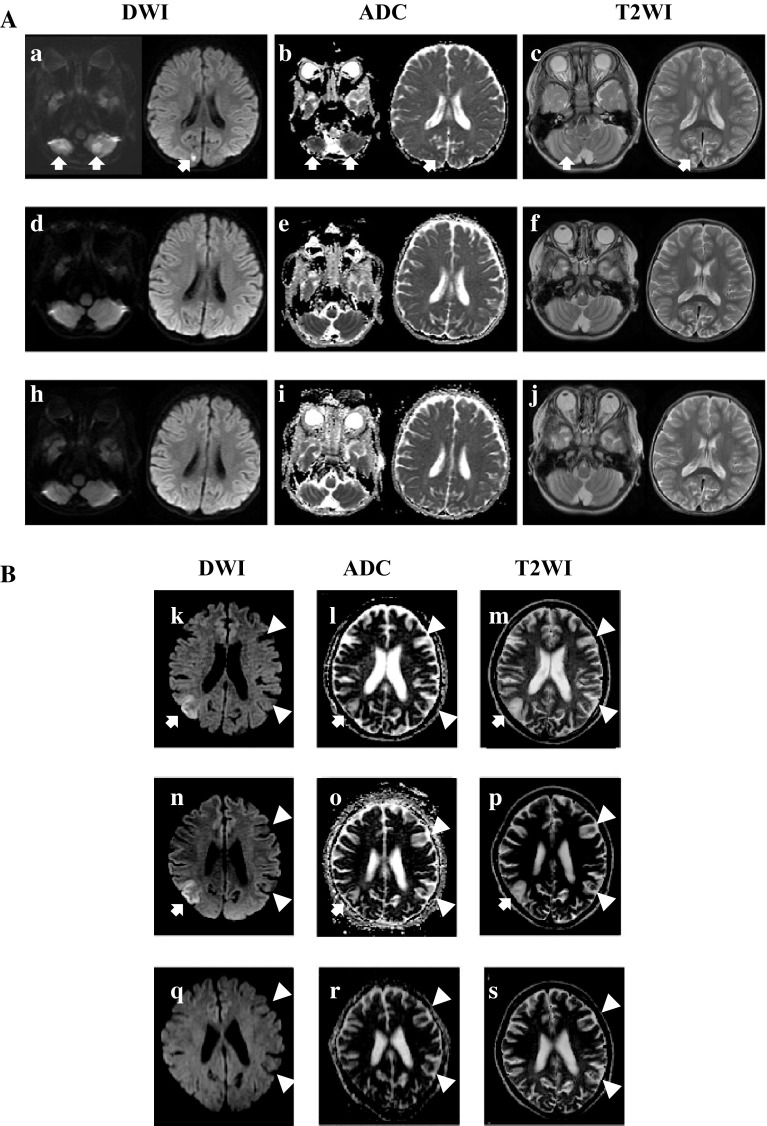
Serial brain MRI images in two MELAS patients. **A** A serial brain MRI was taken at 18 h (*a*–*c*), 7 days (*d*–*f*), and at 1 month (*h*–*J*) after the onset of SLE in patient 1. DWI showed high intensity signal in bilateral cerebellar cortex and right posterior cortex (*arrow*), where is mild-intensity signal in T2WI, and low intensity in ADC. However those showing abnormal intensity in 2 h after onset of SLE showed normal intensity in DWI, ADC and T2WI at 7 days and at 1 month after the episode. **B** A serial brain MRI was taken at 3 h (*k*–*m*), 3 days (*n*–*p*), and at 1 month (*q*–*s*) after the onset of SLE in patient 2. T2WI showed mild high-intensity signal in cortex and subcortical white matter in the right parietal lobe (*m*), which become much less high-intensity signal in 3 days (*p*), and become completely normal in 1 month after the onset of SLE (*s*). DWI showed string-like gyriform-hyperintensity localized in the cerebral cortex (*k*, *n*), which harmonized with low intensity signal on ADC map (*l*, *o*). These data suggested that cytotoxic edema exist in the cortex, on the other hand, vasogenic edema exist in the adjacent white matter. However such MRI abnormalities are not recognized in MRI images at 1 month later from the onset of SLE without atrophic change. ADC values of cortex and subcortical white matter in affected gyrus are 0.73 and 1.53 in 3 h, 0.64 and 1.51 in 3 days, and 0.86, and 0.92 in 1 month after the onset of SLE. ADC values are expressed as 10^−3^ mm^2^/s. High intensity signal in cortex and subcortical white matter in the left frontal and left temporal lobe (*white arrowhead*) (ADC and T2WI) seen in patient 2 was the old region of SLE 2 years ago, which was low intensity signal in DWI. *a*, *d*, *h*, *k*, *n*, and *q* were DWI. *b*, *e*, *i*, *l*, *o* and *r* were ADC. *c*, *f*, *j*, *m*, *p* and *s* were T2WI map

Patient 2 is a 32-year-old girl who fulfilled the diagnostic criteria of MELAS and has a 45 % mutation of A3243G in urine. She has history of two SLE and was then followed as MELAS. She complained of the sudden onset of left-hemi-convulsion, severe headache, and vomit. After getting informed consent, she was infused with 0.5 g/kg/dose of l-arginine at 60 min after the onset, and then the series of brain MRI were taken. Hemi-convulsion was getting worse to generalized convulsion, however the seizures and vomit disappeared within 120 min after l-arginine infusion followed by using anti-convulsants. MRI obtained at 2 and 9 h after the onset showed high intensity on T2WI and DWI and low ADC in right parietal lobe. However those abnormal findings completely normalized in MRI at 1 week and 1 month later as shown in Fig. 1.

Abnormal MRI region in superficial layers of cortex showed low ADC with high intensity on DWI suggested cytotoxic edema. Meanwhile, adjacent subcortical white matter that showed high ADC and high intensity on DWI suggested vasogenic edema. The follow up MRIs in two MELAS patients showed no abnormal signal or atrophic change in 7 days and in 1 month after the onset of SLE. Since MRI abnormality seen in MELAS usually lasting several months after the SLE [5], l-arginine infusion at hyperacute phase may prevents the prolonged change of MRI in our two patients. Though MRI abnormalities seen in our patients cannot be ruled out completely by the results of neuronal hyper-excitability, current episodes are the typical and the same as their previous SLE evident by prolonged changes in MRI. We speculate that l-arginine may protect neuron in cortex from permanent cell death, by improving either regional microcirculation of cerebral blood flow [3, 4], permeability of BBB, or interaction by astrocyte–neuron coupling which controls the cerebral circulation [2, 6].

Our data indicated that l-arginine infusion during the hyperacute phase of SLE in MELAS may significantly improve the symptoms, and also completely protect the progression of neuroimaging change from transient to permanent brain atrophy.
